# Pseudo-Obstruction of the Small Bowel

**Published:** 1969-10

**Authors:** F. Nahai


### PSEUDO-OBSTRUCTION OF THE SMALL BOWEL

**F. Nahai**

The rare condition of pseudo-obstruction of the small bowel was first described by Naish, Capper and Brown in 1960. The main features of the condition are colicky abdominal pain and distension which are due to dilatation of the small bowel; malabsorption with steatorrhoea; episodes of acute intestinal obstruction for which no mechanical cause can be found. The condition is almost certainly due to inco-ordinated peristalsis and there are interesting histological findings.

### CASE REPORT

The patient was a 19 year old female art student who first presented in April 1968 at the Royal Infirmary in Bristol with a 2 year history of abdominal pain and istention, borborygmi and flatulence. Her bowels opened 2 to 3 times a day and her stools were grey in colour. She was losing weight. She also had ankle swelling and complained of dyspnoea on exertion. On examination she was very thin and her secondary sexual characteristics were poorly developed. Her skin was dry and her tongue smooth. Her abdomen was grossly distended and active peristalsis was visible through her thin abdominal wall.

*Investigations*

Relevant investigations before and after treatment are presented in Table I. Radiological investigation showed that she had a normal bone age. A barium meal and follow-through examination showed grossly dilated gas-filled loops of small bowel, the widest lumen being 10 cm. in diameter. In the erect position fluid levels were seen in the dilated bowel. The investigations showed that the patient had gross steatorrhoea but no evidence of coeliac disease or pancreatic insufficiency. It was thought that she had a

TABLE I

**Relevant Investigations**

| | | | | | |
|---|---|---|---|---|---|
| Potassium 3 MEq./L | | | | | |
| Proteins | Total | 4.4 gm% | after | Total | 6.0 gm% |
| | Albumin | 2.8 gm% | treatment | Albumin | 4.0 gm% |
| | Globulin | 1.6 gm% | | Globulin | 2.0 gm% |
| Serum $B_{12}$ 100 uug/ml. ... | | | | | |
| Xylose Absorption Test ... | | | 3.5% of dose excreted in 2 hours<br>11.0% of dose excreted in 5 hours | | |
| after treatment ... ... | | | Normal | | |
| Schilling Test ... ... | | | Negligible excretion of labelled $B_{12}$ | | |
| after treatment ... ... | | | 3.7% of dose excreted | | |
| Fat Balance ... ... | | | Faecal fats 27.0 gm/day | | |
| after treatment ... ... | | | Faecal fats 4.6 gm/day | | |
| Duodenal Juice Tryptic Activity | | | 24 Units (normal $>$ 10) | | |
| Small Bowel Biopsy ... | | | Normal mucosa | | |
| Intestinal Aspirate ... ... | | | Free bile salts demonstrated<br>Culture grew Coliforms | | |

massive blind loop syndome due to a bowel wall lesion. At this stage a diagnostic laparotomy was planned, but in view of the part played by bacteria (in a concentration of $10^6$ organisms per ml.) in producing the symptoms, treatment with ampicillin was started. She responded very well to this, there was symptomatic relief, she started to gain weight and her abnormal biochemical findings returned to normal as seen in Table I.

She was readmitted in December 1968 for diagnostic laparotomy. She was now symptomless, and small intestinal absorption studies were normal. At laparotomy distended flaccid loops of small bowel were seen. Peristalsis was constant but irregular and unco-ordinated. The bowel wall was a dark brown colour because of deposition of the pigment lipofuscin; this is a non-specific change seen in various malabsorptive states. A full-thickness biopsy was taken. The mucosal pattern was normal; there was an increase in the number of plasma cells and lymphocytes in the lamina propria of the mucosa. This would be consistent with bacterial proliferation within the lumen. The striking feature however was the hypertrophy of both the inner circular and outer longitudinal muscle coats. The myenteric plexus between the two muscle layers was hypertrophied. There was Schwann-cell proliferation, and the ganglion cells were large and abnormal.

She was discharged in January after an uneventful post-operative course, but was readmitted two weeks later with a deterioration in her condition. She again had colicky abdominal pain and distention. Culture of her intestinal aspirate gave a profuse growth of coliform bacilli sensitive to "bactrim" and "colistin", so her antibiotic was changed. Unconjugated bile salts were again isolated from the jejunal aspirate. Urinary indoxyl sulphates were estimated and found to be very high. Despite further changes in antibiotics there was little change in her condition though decompression through a naso-gastric tube with intravenous feeding brought some improvement. She had a second laparotomy in February at which the small bowel was decompressed and jejunal and ileal enterostomies were made and a fairly small bore catheter was sutured in position. The post-operative course was uneventful and she was discharged in March. When seen at a recent follow-up she was symptomless and had gained a stone in weight.

### DISCUSSION

A dozen cases of this condition have been reported in the world literature, though it is interesting to note that five of these presented in this region. However the true incidence of the condition is unknown.

Naish *et al.* (1960) collected 8 possible cases of pseudo-obstruction and steatorrhoea from the literature in which some of the features of the syndrome occurred, but in only one case was the complete picture seen.

Dyer *et al.* (1969) point out that the absence of certain features may be due to investigation early in the course of the disease; the condition was not confirmed in their patient until a third laparatomy was done 17 years after the onset of symptoms.

The features of the 5 local cases are presented in Table II. Of these 3 were females and 2 males, ranging in age from 19 to 70 years. Abdominal pain and distention were the main presenting features. All the patients had gross steatorrhoea, though only 3 complained of diarrhoea, Two of the patients complained of vomiting. Small gut histology in all 5 patients showed a normal mucosa, and muscle hypertrophy was present in laparotomy biopsy

TABLE II

| | | (Naish et al) 1960 | (McClelland et al) 1962 | (Pearson et al) 1969 | |
|---|---|---|---|---|---|
| **Age** | 19 | 36 | 55 | 45 | 70 |
| **Sex** | F | M | F | M | F |
| **Presentation** | | | | | |
| Abdo. Dist. | + | + | + | + | — |
| Abdo. Pain | + | + | + | + | + |
| Vomiting | — | + | — | + | — |
| Diarrhoea | — | — | + | + | + |

specimens from 4 patients. The abnormality of the myenteric plexus was only demonstrated in one case. Special histological techniques are required to demonstrate these changes which are best seen in silver preparations cut parallel to the bowel wall as described by Smith (1967a). The apparently normal ganglia seen in two of the patients does not exclude the diagnosis, as conventional techniques may not reveal the lesion.

These changes differ from the neuronal damage caused by *Trypanosoma cruzi* in Chaga's disease and from the changes seen in Hirschprung's disease as pointed out by Smith (1967b). They also differ from aganglionosis of the bowel in which no ganglion cells at all are seen.

The disordered bowel motility is attributable to the myenteric plexus lesion. The smooth muscle hypertrophy results from the abnormal innervation. These changes in turn are responsible for the clinical findings of abdominal pain and distention. The stagnant loop syndrome results from bacterial colonization of these abnormal segments. The evidence for this in the case presented was: (1) the culture of bacteria from the jejunal aspirate, (2) the raised urinary indoxyl sulphates, (3) demonstration of unconjugated bile salts, (4) reduced vitamin B12 absorption, and (5) the response to antibiotics. All these abnormalities returned to normal with treatment.

The duration of symptoms in the 5 patients ranges from 3 to 15 years. This would suggest that the condition is of variable severity; and as the aetiology is unknown treatment is mainly symptomatic.

In view of the part played by bacteria in producing the clinical picture, broad-spectrum antibiotics have been used with some success in three cases. The case presented responded initially to "ampicillin" and the patient reported by Pearson *et al.* (1969) responded very well to "tetracycline". Decompression through a naso-gastric tube relieves the abdominal pain and distention, but this can only be a temporary measure as intravenous feeding is required. We think decompression through enterostomies (as in the patient described) might be used more often in these cases as the third possibility, resection, would seem a drastic measure in a patient who is already suffering from malabsorption. Beyond that one cannot with certainty decide at laparotomy what segment of bowel is affected and there is no guarantee that once the affected segment has been removed the condition will not reappear in the hitherto normal segments of bowel. Resection was undertaken in the patient reported by Dyer *et al.* (1969) in whom the second and third parts of the duodenum, together with the proximal dilated 100 cm. of jejunum, were resected. This resulted in symptomatic improvement.

SUMMARY

A case of pseudo-obstruction of the small bowel is presented. The features of the condition are described and the treatment is outlined. The features of 5 local cases have been summarized. The basic lesion would appear to be an abnormality in the myenteric plexus resulting in disordered peristalsis.

I am very grateful to Dr. A. E. Read for his advice and encouragement in preparing this paper. The patient presented was in hospital under his care.

### References

Dyer, N. H., Dawson, A. M., Smith, B. F. and Todd, I. P. (1969) British Medical Journal *1*, 686.

McClelland, H. A., Lewis, M. J. and Naish, J. M. (1962) Gut *3*, 142.

Naish, J. M., Capper, W. M. and Brown, N. J. (1960) Gut *1*, 62.

Pearson, A. J., Brzechwa-Ajdukiewicz, A. and McCarthy, C. F. (1969) American Journal of Digestive Diseases *14*, 200.

Smith, B. F. (1967a) Gut *8*, 308.

Smith, B. F. (1967b) Journal of Pathology and Bacteriology *94*, 462.

